# Study protocol: a pilot randomized controlled trial to evaluate the acceptability and feasibility of a counseling intervention, delivered by nurses, for those who have attempted self-poisoning in Sri Lanka

**DOI:** 10.1186/s40814-018-0341-1

**Published:** 2018-09-24

**Authors:** A. N. L. M. De Silva, Andrew H. Dawson, Indika B. Gawarammana, Sampath Tennakoon, Thilini Rajapakse

**Affiliations:** 1 South Asian Clinical Toxicology Research Collaboration, Faculty of Medicine, University of Peradeniya, Peradeniya, Sri Lanka; 2 Central Clinical School, University of Sydney, Sydney, Australia; 3 Department of Medicine, Faculty of Medicine, University of Peradeniya, Peradeniya, Sri Lanka; 4 Department of Community Medicine, University of Peradeniya, Peradeniya, Sri Lanka; 5 Department of Psychiatry, Faculty of Medicine, University of Peradeniya, Peradeniya, Sri Lanka

**Keywords:** Non-fatal self-poisoning, Counseling, Nursing

## Abstract

**Background:**

Deliberate self-harm in the form of non-fatal self-poisoning is a major public health problem in Sri Lanka. Previous work suggests that many nurses in Sri Lanka—particularly those who work in primary care in the medical treatment of persons who attempt self-poisoning—already approach their role in a holistic fashion and consider “advising” or “counseling” patients after self-poisoning to be a part of their nursing role. But there is no formal training given to such nurses at present nor has the efficacy or feasibility of such an intervention been assessed in Sri Lanka. The aims of this pilot study are to explore the potential efficacy, acceptability, and feasibility of carrying out a counseling intervention that could be delivered by nurses for persons who present to hospital for medical management of non-fatal self-poisoning.

**Methods/design:**

The study will be carried out at the Toxicology Unit of Teaching Hospital Peradeniya, Sri Lanka. A pilot randomized controlled trial will be carried out among participants admitted to Teaching Hospital Peradeniya for medical management of non-fatal self-poisoning. The primary objective of this study is to explore the acceptability and feasibility of a counseling intervention being delivered by nurses. The secondary objectives are to explore the efficacy of the intervention for the improvement of skills to cope with situations of acute emotional distress, and to reduce rates of anxiety, depression, and future repetition and suicidal ideation. A nurse’s experiences and attitudes regarding the acceptability and feasibility of implementing this intervention and participant experiences of the intervention and its effects will be explored via qualitative interviews and focus group discussions.

**Discussion:**

It is anticipated that the findings of this pilot study will help determine and assess the acceptability and feasibility of this counseling intervention, as well as indicate the more useful aspects of this intervention in order to develop it for further exploration in a larger trial.

**Trial registration:**

SLCTR/2017/008 Registered on 21st March 2017

## Background

Non-fatal self-poisoning is a major health problem in Sri Lanka [1]. Rates of hospitalization due to self-poisoning in Sri Lanka have increased in recent years; from 328 per 100,000 in 2004 to 463 per 100,000 in 2010 for males and from 270 to 421 per 100,000 with respect to females [2]. Recent studies indicate that there is a rise in medicinal overdoses in both urban and rural areas [3, 4]. Rates of self-poisoning are also high in Sri Lanka, Bangladesh, and India when compared with other South Asian low and middle-income countries (LMIC) [5].

Many terms—such as deliberate self-harm (DSH), attempted suicide, or attempted self-poisoning— are used to describe the behavior of those who deliberately ingest a poison or overdose a medication. For the purpose of this study, we use the term non-fatal self-poisoning, defined as deliberate ingestion of a toxic substance or ingestion of a medication in excess of its prescribed dosage.

Non-fatal self-poisoning in Sri Lanka appears to be associated with a combination of socio-demographic, cultural, and psychological factors [6]. The interpersonal conflict appears to be a common factor immediately preceding the act of non-fatal self-poisoning for both males and females, and the act occurs usually with only a small period of premeditation [7, 8]. Marecek et al., in their examination of self-poisoning by females in Sri Lanka, report that the act of self-poisoning is often associated with an interpersonal dispute, most often related to issues regarding the girl’s comportment and heterosexual relations [8]. Other studies describe a mixture of motivations associated with the act of self-poisoning, including a desire to die, a desire to escape, and difficulty tolerating distressing emotion associated with interpersonal conflict [7]. Factors such as physical, sexual, or psychological abuse and living in economically or psychologically stressful circumstances are also likely to contribute towards this behavior at times of crisis [9]. Particularly among older persons, psychiatric morbidity such as depression and alcohol use disorder in males is also likely to contribute [10, 11]. Lower socioeconomic status, unemployment, and lower levels of education have also been associated with self-poisoning [6, 12]. Stressful life events such as failed harvest, harassment at school, failed examination, and financial stressors are reported associated factors [9, 13].

### Psychological interventions for minimization of non-fatal self-poisoning

Internationally, many different types of psychological interventions have been explored to minimize this issue with varying results. Some interventions have been directed towards all patients presenting with non-fatal self-poisoning. Certain interventions have targeted specific subgroups—such as those suffering from depression or borderline personality disorder, specific ethnic groups, adolescents, or females and youth [14–17]. These include interventions based on cognitive behavior therapy, problem-solving therapy, family interventions, interpersonal therapy, and other innovative methods such as follow-up contact by telephone and postcards and short message services (SMS) [17–25].

However, studies reporting on interventions for DSH in non-western countries are scarce [24]. A study from Pakistan has reported that a culturally adapted, manual-assisted problem-solving therapy (C-MAP) was effective in reducing suicidal ideation and in improving quality of life and coping skills following self-harm [26]. This was a manual-assisted intervention, adapted from a self-help guide based on the principles of cognitive behavior therapy (CBT), and delivered by qualified psychologists.

Published interventional research for prevention of non-fatal self-poisoning in the Sri Lankan context is limited. One study conducted among young people in an urban population in Sri Lanka suggested that problem-solving counseling may be an effective and culturally acceptable therapeutic tool in the secondary prevention of suicides, which improved coping skills in the target group [27]. Another pilot has suggested that vendor-based sales restriction in regions with high rates of self-poisoning has the potential to reduce access to pesticides for self-poisoning in the Sri Lankan context [28]. However, there are only a few published data available regarding interventions for prevention of non-fatal self-poisoning in rural Sri Lanka, despite the widespread nature of the problem. Therefore, exploring interventions that are acceptable and feasible to carry out in a Sri Lankan social and cultural context is of importance.

### Nurses in mental health and counseling

Previous studies have shown that nurses indeed do engage in counseling of patients informally and without specific training in different community environments [29, 30]. A survey conducted by the World Health Organization (WHO) and the International Council of Nursing about nurses in mental health confirms that nurses are the central providers of most mental health services in many LMICs, but often with inadequate levels of training [31, 32]. Studies from Sri Lanka have shown that nurses also engage in providing mental health services in different communities. A recent study about nurses’ role in caring for and supporting women experiencing intimate partner violence in Sri Lanka has reported that nurses provide various kinds of support, including psychological support and advice to abused women [33]. Another study has shown that rural nurses who care for patients following non-fatal self-poisoning in rural Sri Lankan communities have a high level of first-hand knowledge of the patient’s life stressors and show understanding of how these interconnected and contributed as direct and indirect risk factors for self-poisoning [13].

Given the increasing rates of hospital admissions for non-fatal self-poisoning in Sri Lanka and the association with triggers such as interpersonal conflict, it is well worth exploring whether similar psychological interventions would be efficacious in improving coping skills. Such a psychological intervention would need to be adapted to the local culture and sustainable in the long term; while the field of mental health has developed greatly in Sri Lanka in recent years, an interventional therapy that requires large input from psychologists or even psychiatrists for its delivery is unlikely to be sustainable in the long-term, given how widespread the phenomena of self-poisoning in Sri Lanka is at present, combined with the current socio-economic constraints and limitations in trained resource personnel.

Thus, the primary objective of this study is to explore the acceptability and feasibility of a counseling intervention being delivered by nurses. The secondary objective is to explore the potential efficacy of the intervention for the improvement of skills to cope with situations of acute emotional distress and to reduce rates of anxiety, depression, and future repetition and suicidal ideation. It is anticipated that the findings of this pilot study will help determine and assess the acceptability and feasibility of this counseling intervention, as well as indicate the more useful aspects of this intervention in order to develop it for further exploration in a larger trial. It will also inform other aspects of a future trial, such as the sample size, indicate the level of loss to follow-up, and help assess treatment as usual.

## Methods/design

**Fig. 1:**
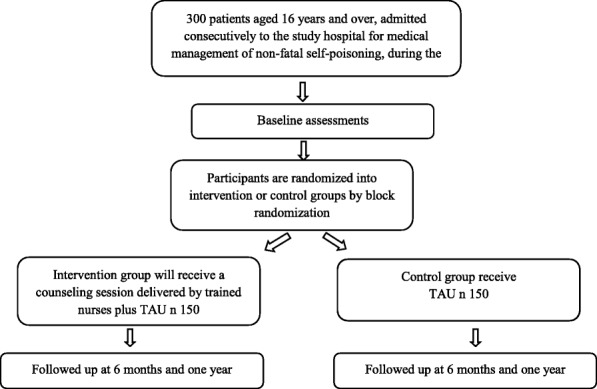
Study flow chart

This will be a pilot randomized controlled trial (RCT) to evaluate and explore the efficacy of a counseling intervention delivered by nurses for persons who have been admitted to hospital for medical management of non-fatal self-poisoning (Fig. 1).

Primary outcomes will be assessment of feasibility and acceptability of the counseling intervention delivered by nurses. Acceptability and feasibility of this intervention, as experienced by the nurses who deliver the intervention and the participants who receive the intervention, will be assessed qualitatively. The secondary outcome is to explore the potential efficacy of the intervention for the improvement of skills to cope with situations of acute emotional distress and to reduce the rates of anxiety, depression, and future repetition and suicidal ideation.

### Study setting

The study will be carried out at Teaching Hospital, Peradeniya. Teaching Hospital Peradeniya is a large tertiary care teaching hospital in the Central Province of Sri Lanka. Most patients who attempt self-poisoning in the Central Province and surrounding regions, and who require medical care, are admitted for medical management to the Toxicology Unit (Ward 17) of this hospital [34].

### Phase 1—Randomized controlled trial

#### Inclusion criteria

Persons aged 16 years and over admitted to the study hospital for medical management of non-fatal self-poisoning during the study period, will be considered eligible for the participation in the study. Persons with first, as well as repeat, attempts of self-poisoning will be considered eligible for inclusion in the study. Further, we will be including bilingual persons who are fluent in speaking in Sinhala as well.

#### Exclusion criteria

Persons who state or carry reports indicating a previous diagnosis of mental retardation or dementia, and those who are physically too unwell to participate in the interview, will be excluded. Those who are not fluent in spoken Sinhalese will also be excluded from the study.

Potential participants will be provided verbal and written information about the study, and those who give written informed consent will be recruited to the study. The principal investigator will talk to potential participants and obtain informed consent.

### Enrollment and randomization

Participants who meet the inclusion criteria as described above, who have given written informed consent, will be randomly allocated (concealed) to either the intervention arm or the control arm. Those randomized to the intervention arm will receive the intervention in the form of a counseling session by a trained nurse. Both the intervention and control groups will receive medical care and treatment as usual (TAU) during their hospital stay. This will include medical management as well as psychiatry input for all irrespective of the intervention/control status of the participant. Care will also be taken to ensure that the intervention does not interfere with any treatment as usual.

Recruitment and intervention will be conducted over a consecutive 5-month period, or until the required number of participants are enrolled, and the end date of the study will be 12 months after the baseline intervention.

#### Randomization procedure

The intervention will be assessed using block randomization technique, in order to reduce bias and achieve balance in the allocation of participants to the intervention and control arm. An allocation sequence will be provided by the off-site statistician (independent of the research team) and will be based on a computer-generated list of random numbers, to allocate patients to the intervention plus TAU or TAU alone.

#### Description and delivery of the trial intervention

The intervention consists of a brief single-structured counseling intervention delivered by trained nurses. It has been developed based on a similar intervention for self-harm described from Pakistan (C-MAPS) [26]. The C-MAP is a brief problem-focused therapy, conducted in six sessions over 3 months [26]. The aim of the original C-MAP intervention is to help the participant identify and resolve interpersonal difficulties, which cause or exacerbate distress. Previous work in Sri Lanka and South Asia have reported the common finding that self-harm is often associated with recent interpersonal conflict and associated with acute emotional distress (against a background of more complex factors), and the act is often carried out with a mixture of varied motives. Shortly after the act, the distress abates; but such persons have reported marked distress and mixed motivations at the time of the attempt—such as the desire to escape unbearable pain/thoughts/situation, inability to think of anything else to do, the wish to communicate, anger, shame, etc.

Therefore, in the current study, we have chosen to focus on the aspect of coping with the “acute emotional distress” associated with interpersonal conflict, and to do so, we have adapted the C-MAP therapy which has been developed and used in Pakistan [26]. This intervention focuses on acknowledging this distress and exploring alternate simple ways that suit participants to cope with such acute distress in the future. In this way, the nurses delivering the intervention will be encouraged and trained to, as far as possible, elicit ways of facing future crisis and negative emotions, from the person receiving the counseling himself/herself, and to then discuss and emphasis these strategies further.

The original C-MAP included sessions on introduction, dealing with crisis and problems, changing thoughts, and dealing with family problems. Some of these steps will be kept as the focus of this intervention as well—with a particular focus on dealing with crisis situations causing acute emotional distress [26]. But having multiple intervention sessions, which require the participants to return to the hospital after discharge, is unlikely to be practical in the context of this study—the participant attrition rate is likely to be high, and the demand on the nurses’ time, is also likely to be too much. And although the nurses will receive a training on delivering the counseling intervention, they will not have the expertise to deliver complex psychological therapies. Therefore, considering these factors, the intervention in this study will be limited to one session, delivered by nurses, prior to discharge from the hospital. Considering that the intervention is to be limited to one session and that the nurses do not have formal training in psychological therapies, the “changing thoughts” part of the original C-MAP therapy will be omitted from this intervention. Thus, the intervention in this study focuses more on identification and acknowledgement of problems or difficulties and exploration of alternate ways to cope with such situations of acute emotional distress in the future; this would include discussion of strategies such as emotional ventilation, relaxation, distraction, or self-soothing—guided by participant preferences as well.

The intervention will be carried out as a single conversational session, between a trained nursing officer and the participant. Each session will last about 30 min. It will be carried out in a confidential setting during hospitalization, within 1 week of the self-harm attempt (after the acute phase of the hospitalization when the participant has received medical treatment and management of the poisoning and its physical manifestations, but prior to discharge). At the end of the discussion, the participant will be given a brief, “take-home message’,” written in the form of a personalized note on the back of a pocket calendar, which sums up the main conclusions discussed by the nurse and the participant. The participant will be encouraged to keep the pocket calendar and message as a reminder of alternate ways of coping with distress; care will be taken, however, to write the message in a de-identified form, without any direct reference to the incident of self-poisoning, and without revealing any confidential information.

At the start of the RCT, the nurses delivering the intervention will receive a specific training, coordinated by the researcher, regarding the nature of the intervention as described above. This training will be conducted in the form of interactive workshops, conducted by the investigators, and will include demonstrations and role play. The training will include a basic introduction into what is meant by counseling, followed by training about the specific counseling required in this study. This will include aspects such as introduction, building up a rapport, listening to the participant, reflecting back, helping the person himself/herself identify the problem or trigger that led up to the act of self-poisoning, acknowledgment of the associated emotional distress, and exploration of alternate ways to deal with situations of acute emotional distress should it occur again in future. The need for maintenance of privacy and confidentiality will be stressed. There will be also a focus and discussion on limits and boundaries and identification of risks, as well as when and how to refer for further help. The training will include workshops and role play, and nurses will also be given a structured manual which outlines the principles of the counseling intervention, and they will be encouraged to use this as a guide when delivering the intervention.

During the nurses training, all attempts will be made to build confidence and provide a positive background towards carrying out this role in the ward. Possible challenges the nurses may face, such as time constraints, and other issues will be looked for and discussed during these workshops. Nurses will be encouraged to carry out the counseling at a convenient time. It will also be made very clear that the nurse’s participation and contribution towards this intervention are entirely voluntary. The nurses will also be given a structured manual which outlines the principles of the counseling intervention, and they will be encouraged to use this as a guide when delivering the intervention.

There will be an ongoing contact between the nurses involved in the study and the researcher to monitor for any difficulties during the implementation of the intervention. Before starting the intervention, the investigators will provide a description about the background and rationale of doing this study to other health staff who will be working at the study site to enlist their support.

All participants (intervention and control group) will receive medical care and treatment as usual during their hospital stay. This will include medical management as well as psychiatry referral, as deemed required by the medical team (which is the usual practice) irrespective of the case/control status of the participant. Care will also be taken to ensure that the intervention does not interfere with any treatment as usual.

#### Delivery of the intervention

The intervention will be delivered by one or two designated trained nursing officers who have an interest in this new role and are willing to deliver the intervention. The details of the nurses training have been described previously.

### Outcome measures

#### Baseline assessment

Qualitative interviews will be conducted with participants, as described in the “Phase 2—Qualitative study” section below. The following assessments will also be completed among all participants: assessment of level of suicidal intent associated with the recent act of non-fatal self-poisoning (via the Pierce Suicide Intent Scale (PSIS), an internationally used scale for the assessment of suicide intent that has been used previously in Sri Lanka [35]) and presence of depression (via the Patient Health Questionaire-9 (PHQ-9) [36, 37] and Peradeniya Depression Scale (PDS) [38]). The PHQ-9 is a well-recognized tool for the screening of depression and has been validated for use in Sri Lanka, with the cutoff score of 10 or above indicating the presence of depression [38]. The PHQ-9 can also be used to examine the severity of depressive symptoms, with scores of 1–4, 5–9, 10–14, 15–19, and 20–27, indicating minimal, mild, moderate, moderately severe, and severe depressive symptoms, respectively [38]). The PDS is a screening tool for the identification of depression that was developed and validated for use in Sri Lanka, which considers the local cultural idioms of distress [38]. Participants will also be screened for anxiety (via the Generalized Anxiety Disorder 7-item scale (GAD 7)) [39] and psychological distress (via the General Health Questionnaire (GHQ 30)) [40] and assessed for coping strategies (via the Brief Coping Inventory, Brief COPE [41]). As far as possible, scales that have been translated to the local language and validated for use in Sri Lanka will be used. In the case of the GAD 7 and the Brief COPE, no validated versions are available, but these scales will be translated to Sinhala and back-translated and checked for accuracy prior to use. The baseline investigations will be carried out a few days (up to a week) after the act, allowing time for recovery from the immediate distress following the act of non-fatal self-poisoning.

### Follow-up assessments at 6 months (intermediate) and 1 year (final assessment)

Qualitative interviews will be conducted among the participants to explore the acceptability of the counseling intervention, whether the participants perceived it as helpful, particularly with regards to coping with acute emotional distress, and components (if any) of the intervention that they particularly remembered or liked. Details of the qualitative assessments are given below (phase 2). Nurses who delivered the intervention will also be interviewed regarding their attitudes and perceptions towards the acceptability and feasibility of delivering the intervention (further details are provided below, in the “Phase 2—Qualitative study” section).

Additionally, assessment of levels of anxiety, depression, and how the person copes with situations of acute emotional distress will be conducted using the measures described above for the baseline assessment. Further secondary outcomes of interest will be rates of repetition and the presence of suicidal ideation, at 6 months and 1 year follow-up. This will be assessed using a structured interviewer-administered questionnaire.

The follow-up assessments at 6 months and 12 months will occur at the hospitals where the initial recruitment took place. Care will be taken to conduct the 6-month and 1-year assessments in a confidential and sensitive manner, to avoid any further distress. After the final assessment, the participants will be informed that this is the last study assessment, but if relevant, they will be encouraged to continue treatment as usual as instructed by their treating team. If required (if the participants are found to require further psychiatric care during the 6 months and 1 year follow-up), the participants will be linked with and referred to ongoing psychiatric care and follow-up.

### Plan of contact for those who do not attend

All participants will be reminded of their follow-up assessment by a telephone call and letter, prior to their scheduled follow-up visit. They will also be offered re-imbursement to cover their travel costs to the hospital. Those who do not attend their follow-up visits (either the intermediate or final evaluation) will be contacted again by telephone, and by letter, and will be encouraged to attend. If the person does not respond to two phone calls and the written reminder, they will be considered lost to follow-up.

#### Sample size

Previous interventional studies have examined for changes in the severity of depression, using the PHQ-9 as an outcome measure [42]. One study reported that patients in the intervention group had significantly fewer depressive symptoms compared with patients in the usual care group at 12 weeks follow-up, and the PHQ-9 mean score at 3 months follow-up was 2.4 and 7.1 in the intervention and control group respectively (4.7 mean difference) [42]. The difference between the groups was significant (*p* < .001) using baseline scores as the covariate. A sample size of 15 in each group gave an 80% power to detect the difference in means of 4.7, assuming that the estimated common standard deviation is 4.49 and using a two-group *t* test with a two-sided significance level of 0.05 [42]. Based on similar calculations and allowing for a 50% drop-out rate, we calculated a minimum of 30 participants in each group; however, since our study will be conducted among those who have attempted self-poisoning, we anticipate (based on previous studies) that a little less than 50% of the sample will be suffering from depression [43]. Furthermore, through this intervention, we are planning to examine for clinically relevant changes of depression levels (PHQ-9 scores above 10, indicating a moderate level of depression or above, rather than in the < 10 range quoted in the previous study). To allow for this greater margin, we plan to recruit a sample of 150 intervention and controls.

### Phase 2—Qualitative study

The qualitative component of this study complements the quantitative component of the study. Using qualitative methods, we will explore the feasibility and acceptability of the newly introduced intervention in a large tertiary care hospital setting, from the point of the nurses as well as those who receive the intervention, and will explore a range of perspectives.

We will conduct qualitative in-depth interviews with the participants during the hospital stay (pre-intervention) and follow-up (post-intervention). This is a central part of the qualitative study as we consider the experiences of nurses who deliver the intervention and participants to be crucial to evaluate the acceptability and feasibility of the counseling intervention. Further, we recognized that the screening instruments using in the quantitative study may not fully capture the complex interplay of factors that may contribute towards the act of self-poisoning. The qualitative component of the study will help explore the social and cultural aspects related to non-fatal self-poisoning which could be effective to develop future interventions to reduce non-fatal self-poisoning in Sri Lanka. These interviews will be also conducted with a view of the triangulation of qualitative and quantitative data.

### Interviews with nurses who deliver the intervention

In-depth interviews will be conducted with the two designated trained nurses who voluntarily engaged to delivered the intervention, soon after the completion of the intervention period (of the RCT), to explore feasibility and acceptability of the intervention, as perceived by the nursing officers. The interviews will allow them to share stories and challenges broadly in an open approach. The nurses’ overall experiences regarding their new role of counseling persons who have attempted self-poisoning will be explored. This will include their perceptions of the experiences of delivering the intervention—whether they thought the training was adequate—use of the manual, what skills they think are needed for being an effective counselor, impact of age or service experiences of nurses on delivering the intervention, and challenges they faced and their suggestions for further improving the process. This will be conducted allowing the maintenance of an open approach so that the participants can bring in their own perspectives.

#### Interviews with study participants

Similarly, qualitative interviews will be conducted among the study participants who received counseling from the nurses, as well as those who did not receive the counseling intervention, during the hospital stay and at 6 months follow-up. Exploring perceptions, experiences, and attitudes of the participants about the counseling they received from the nurses is the key in order to explore the acceptability of the intervention from the recipients’ point of view. We will also explore their attitudes and perception towards the counseling intervention they received and whether they found it helpful or unhelpful with regards to coping with situations of emotional distress. These interviews will also explore the characteristics of nurses as counselors that participants identify as supportive or unsupportive and to see whether this can be taken into consideration to the further improvement of the intervention.

We will also explore participant experiences after the act; the factors or reasons for the act as perceived by the participants and their family reaction towards the act. Further interviews will also be used to explore the way participants perceive how they cope with situations of acute emotional distress pre- and post-intervention.

The interviews will be semi-structured, guided with a theme list to cover all relevant issues during the interview, and will be conducted in Sinhala in a culturally sensitive manner. It is anticipated that the interviews will encourage participants to share sensitive issues and stories relating to the self-poisoning incident.

Participants will be selected following maximum variation sampling strategy technique [44], representing different age groups, employment groups, ethnic groups, and rural/ urban origin for the interviews, and interviews will be conducted until data saturates.

### Data management

Data collection and accurate documentation will be the responsibility of the study staff under the supervision of the principal investigator. The principal investigator, other investigators, and research coordinator will be the involved research staff in this study. All source documents will be reviewed by the study team and data entry staff who will ensure that they are accurate and complete. All interviews and focus group discussion will be audio recorded and transcribed in the original Sinhala language by the researcher. All the data and transcriptions will be kept strictly confidential and only referred to by the researcher.

### Analysis

A strict intention to treat approach will be followed in the analysis. For missing data with regard to the main outcome, variable imputation will be used. The study and the control groups will be described using descriptive summary statistics (mean, standard deviation, count percent). The effect of the intervention will be assessed with a relative risk of severity of depression and other endpoints such as coping skills, levels of anxiety, depression, alcohol misuse, and repetition of self-harm.

Analyzing the qualitative interviews will be started parallel to the conducting of the interviews. The researcher will read the interview transcripts carefully several times to have a sense of the whole data, identifying emerging themes and categories [45]. She will discuss emerging themes, concepts, and contradictions with other designated investigators in the study. A code book will be created and modified until the final set of codes is obtained and reached to the data saturation point when there is no emergence of further new themes.

Qualitative data analysis will be conducted using a thematic content analysis approach following a systematic process. Codes will be applied to the transcripts and converted into categories to represent the main themes arising from the data [46]. Triangulation of themes and concepts will be used to further compare and contrast the data from the different participating groups. It is anticipated that this will strengthen the reliability and validity of the analysis. The analysis will be conducted in the original Sinhala language, and final themes will be translated into the English language by the researcher in cooperation with a professional translator.

## Discussion

This paper describes the protocol for a pilot study that explores the feasibility and acceptability of a nurse-delivered counseling intervention for those who have been admitted to the hospital for medical management of non-fatal self-poisoning. There will be a focus on the exploration of nurses’ perceptions of the intervention, as well as participants’ perception of the acceptability of the intervention, and whether they find it helpful in coping with acute emotional distress in alternate ways. We will be using mixed methods to enhance of the richness of the findings and to explore in depth what aspects of the intervention (if any) the participants find more useful, and it is anticipated that these findings will inform the further development and refinement of the intervention for future trials. Secondary objectives explore the efficacy of this intervention in dealing with acute emotional distress, as well as reducing depression, future self-poisoning, and suicidal ideation among those who have attempted self-poisoning.

Research indicates increasing rates of non-fatal self-poisoning in Sri Lanka in recent years. Internationally, many different types of psychological interventions delivered by non-specialized health staff in LMIC settings have been explored to minimize self-harm. But to the best of our knowledge, there have been no such trials of low-cost interventions for the prevention of self-harm delivered by non-specialized health staff in Sri Lanka. This is an important aspect to consider in a resource-limited setting such as Sri Lanka.

In this feasibility trial, we have combined two concepts: one is the concept of a counseling intervention for participants to identify situations of emotional distress and combined together with the concept of delivery of the intervention by nursing officers to enhance the use of existing staff, reduce cost, and improve sustainability and acceptability of the intervention.

It is anticipated that assigning nurses as counselors will be more effective, since nurses are more available and accessible for patients compared to psychiatry staff and other mental health services in hospital settings. The patients may also feel more comfortable talking to nurses, and the associated stigma is likely too much less compared to a “psychiatry referral”. However, there are also likely be challenges, such as time constraints, practical difficulties, and negative attitudes on the part of nursing officers, which may act as barriers towards nurses delivering this type of a counseling intervention. Thus, an important aspect of this pilot study is to evaluate the acceptability and feasibility of the intervention, both on the part of the nurses delivering the counseling, as well as the participants receiving it. We also anticipate that by piloting the intervention, we will be able to gain a better understanding of the components of the intervention (if any) that are perceived as more useful or acceptable by the recipients. By using qualitative methods parallel to the pilot RCT, we hope to gather a richer and more comprehensive understanding of these aspects of the intervention.

### Limitations

The screening instruments may not fully capture the complex interplay of factors that may contribute towards the act of self-poisoning, and this is a limitation. However, to minimize this as far as possible, we have opted to use mixed methods for this study—in-depth interviews will be conducted with the participants during the hospital stay (pre-intervention) and follow-up (post-intervention). These interviews will explore varied aspects such as the participant’s perceived associated reasons or triggers for the act, their experiences after the act, and their family reactions towards the act. Qualitative methods will also be used to assess the way participants perceive how they cope with situations of acute emotional distress pre- and post-intervention.

Regarding the developing of the intervention of this study, detailed therapy of “changing thoughts” are not included in this intervention given the limitations. We acknowledge that family interventions/therapy would be beneficial, but again, given the brief and limited nature of the intervention, it could not be included in this study. Further, the intervention in this study will be limited to a single session considering the limitations of cost and feasibility.

## Conclusions

It is anticipated that the findings of this pilot study will help determine and assess the acceptability and feasibility of this counseling intervention, as well as indicate the more useful aspects of this intervention in order to develop it for further exploration in a larger trial.

## Abbreviations

CBT: Cognitive-behavior therapy
C-MAP: Culturally adapted manualized problem solving
GAD 7: Generalized Anxiety Disorder 7-item scale
GHQ 30: General Health Questionnaire
PDS: Peradeniya Depression Scale
PHQ-9: Patient Health Questionnaire
PSIS: Pierce Suicide Intent Scale
RCT: Randomized controlled trial
TAU: Treatment as usual
WHO: World Health Organization
